# Association of Prognostic Nutritional Index with Severity and Mortality of Hospitalized Patients with COVID-19: A Systematic Review and Meta-Analysis

**DOI:** 10.3390/diagnostics12071515

**Published:** 2022-06-21

**Authors:** Kuo-Chuan Hung, Ching-Chung Ko, Li-Kai Wang, Ping-Hsin Liu, I-Wen Chen, Yen-Ta Huang, Cheuk-Kwan Sun

**Affiliations:** 1 Department of Anesthesiology, Chi Mei Medical Center, Tainan City 71004, Taiwan; ed102605@gmail.com (K.-C.H.); anesth@gmail.com (L.-K.W.); 2 Department of Hospital and Health Care Administration, College of Recreation and Health Management, Chia Nan University of Pharmacy and Science, Tainan City 71710, Taiwan; 3 Department of Medical Imaging, Chi Mei Medical Center, Tainan City 71004, Taiwan; kocc0729@gmail.com; 4 Department of Health and Nutrition, Chia Nan University of Pharmacy and Science, Tainan City 71710, Taiwan; 5 Institute of Biomedical Sciences, National Sun Yat-sen University, Kaohsiung City 80424, Taiwan; 6 Department of Anesthesiology, E-Da Hospital, Kaohsiung City 82445, Taiwan; neoplasmboy@yahoo.com.tw; 7 Department of Anesthesiology, Chi Mei Hospital, Liouying, Tainan City 710402, Taiwan; 8 Department of Surgery, National Cheng Kung University Hospital, College of Medicine, National Cheng Kung University, Tainan City 70101, Taiwan; 9 Department of Emergency Medicine, E-Da Hospital, Kaohsiung City 82445, Taiwan; 10 College of Medicine, I-Shou University, Kaohsiung City 84001, Taiwan

**Keywords:** prognostic nutritional index, coronavirus disease 2019, mortality, disease severity, area under curve

## Abstract

The associations of prognostic nutritional index (PNI) with disease severity and mortality in patients with coronavirus disease 2019 (COVID-19) remain unclear. Electronic databases, including MEDLINE, EMBASE, Google scholar, and Cochrane Library, were searched from inception to 10 May 2022. The associations of PNI with risk of mortality (primary outcome) and disease severity (secondary outcome) were investigated. Merged results from meta-analysis of 13 retrospective studies (4204 patients) published between 2020 and 2022 revealed a lower PNI among patients in the mortality group [mean difference (MD): −8.65, *p* < 0.001] or severity group (MD: −5.19, *p* < 0.001) compared to those in the non-mortality or non-severity groups. A per-point increase in PNI was associated with a reduced risk of mortality [odds ratio (OR) = 0.84, 95% CI: 0.79 to 0.9, *p* < 0.001, I^2^ = 67.3%, seven studies] and disease severity (OR = 0.84, 95% CI: 0.77 to 0.92, *p* < 0.001, I^2^ = 83%, five studies). The pooled diagnostic analysis of mortality yielded a sensitivity of 0.76, specificity of 0.71, and area under curve (AUC) of 0.79. Regarding the prediction of disease severity, the sensitivity, specificity, and AUC were 0.8, 0.61, and 0.65, respectively. In conclusion, this study demonstrated a negative association between PNI and prognosis of COVID-19. Further large-scale trials are warranted to support our findings.

## 1. Introduction

The outbreak of the coronavirus disease 2019 (COVID-19) pandemic since the end of 2019 has already claimed millions of lives, as well as imposed enormous threats to healthcare systems and economies worldwide [1]. Despite mild symptoms in the majority of patients contracting the disease, a significant population still experienced severe symptoms and later developed life-threatening complications including acute respiratory distress syndrome (ARDS), pneumonia, and multiple organ failure [2,3]. Of those diagnosed with COVID-19, nearly one-fourth (22.5%) present with severe illnesses and nearly 6% may succumb to the disease [4]. Previous studies have reported a number of risk factors for disease progression, including an advanced age, male gender, and comorbidities [4,5]. Moreover, a recent meta-analysis of 77 observational studies recruiting over thirteen thousand participants has identified the circulating level of cytokines (i.e., IL-6) as a potential predictor of the severity and mortality of those contracting COVID-19 [6]. The finding is consistent with the pathogenesis of COVID-19 that involves uncontrollable immune reactions presenting as inflammatory responses [7,8]. However, the epidemiological characteristics and the risk factors associated with unfavorable outcomes remain to be elucidated [9]. Clarification of these issues may enable effective resource allocation and timely implementation of appropriate treatment strategies [10].

Previous studies have demonstrated an association between a poor nutritional status and an elevated risk of in-hospital fatality in patients infected with COVID-19 [11,12]. Prognostic nutritional index (PNI), which can be simply calculated from albumin concentration and lymphocyte count from peripheral blood, has been demonstrated to be significantly associated with the incidence of postoperative complications and mortality rate in patients diagnosed with various gastrointestinal malignancies [13,14]. Furthermore, PNI has been identified as a simple and reliable prognostic biomarker in other subgroups of patients with acute respiratory disorders, such as the acute exacerbation of chronic obstructive pulmonary disease [15,16]. Taking into account the important roles of immune responses and the nutritional status of the host in COVID-19 progression [17,18], the role of PNI in predicting the severity and mortality of COVID-19 has been studied and validated in previous investigations [19,20,21]. Nevertheless, the prognostic value of PNI from previous observational studies recruiting patients with a different gender prevalence as well as variations in mortality rate and geographical locations based on a single hospital setting remains questionable. Therefore, the present systematic review and meta-analysis aims at evaluating the prognostic value of PNI for predicting the severity and mortality in patients diagnosed with COVID-19.

## 2. Materials and Methods

We report this systematic review in compliance with the Preferred Reporting Items for Systematic Reviews and Meta-analysis statement. Our study protocol has been registered in the International Prospective Register of Systematic Review [CRD42022331321]. For improving the quality of the current meta-analysis, study selection, data collection, and risk of bias assessment were independently executed by two authors. All disagreements were settled by discussion. The protocol and procedures of the current meta-analysis has been described in our previous study [22].

### 2.1. Data Sources and Searches

Using different combinations of keywords and MeSH terms, we searched on 10 May 2022 for studies reporting the association between PNI and prognostic outcomes in adult patients diagnosed with COVID-19 from the following databases: Medline, Embase, and Cochrane Library. Additionally, a manual search was performed in Google scholar to identify relevant articles. Supplemental Table S1 depicts the process of the literature search, taking Medline as an example. There was no restriction on language, year of publication, and sample size when conducting the database search. For a completeness of our search, we examined the reference lists of the acquired articles and published meta-analyses to further retrieve eligible studies for the current investigation.

### 2.2. Study Selection and Data Extraction

We included studies that fulfilled the following criteria: (1) observational studies including cross-sectional, cohort study, and case-control study designs, (2) adult patients who were diagnosed with COVID-19 and admitted to hospital, (3) available PNI values at hospital admission, (4) reporting of the association of PNI values with disease severity or mortality, and (5) studies with adequate details for the calculation or extraction of individual odds ratio (OR) as well as corresponding 95% confidence intervals (CIs). We excluded studies that were (1) duplicated, (2) conducted in the pediatric population or those not admitted to hospital, (3) presented as conference abstracts, animal studies, systematic reviews, case reports, and editorials or commentaries as well as different forms of publication other than the original investigation.

### 2.3. Data Extraction

The following items were retrieved from each study: first author/publication year, age, male gender, number of patients, prognostic outcomes (i.e., mortality and disease severity), PNI values, sensitivity/specificity, negative predictive value (NPV), positive predictive value (PPV), and country. We acquired the odds ratio (OR) and 95% confidence intervals (CI) from matched or adjusted data for each study. For studies that provided both unadjusted and adjusted ORs, we adopted the adjusted ORs. On encountering a categorical variable from the dichotomization of a continuous variable, we calculated the OR by the number of cases and controls with exposure to the prognostic factors (e.g., PNI) according to the cut-off point reported in that study. The authors of our included articles with missing data were contacted for additional information.

### 2.4. Outcomes and Definitions

Our primary outcome was the correlation between PNI and the all-cause mortality during hospitalization, while the association of PNI with disease severity and the diagnostic efficacy of PNI for mortality and disease severity served as the secondary outcomes. The definition of disease severity was based on that in each study. Investigations into the associations of other risk factors (e.g., age and biomarkers) with prognostic outcomes, which were hampered by the limited number of available studies, were not performed in the current meta-analysis.

### 2.5. Assessment of Risks of Bias for the Included Studies

The risk of bias for each study was independently reviewed by two authors in accordance with the six domains of the Quality in Prognostic Studies (QUIPS) tool, namely, study participation, outcome measurement, study attrition, prognostic factor measurement, adjustment for other prognostic factors, and statistical analysis and reporting [23]. For each domain, the risk of a study was classified as low, unclear, or high. The overall risk of bias of a study was deemed low when all or most of the domains were judged to be low (or low to moderate) in that study [24].

### 2.6. Data Synthesis and Analysis

To assess the association between PNI and the prognostic outcomes, three different approaches were applied. First, the preoperative PNI was compared between the survival and non-survival groups; second, the association between PNI and the risk of mortality was investigated, with the PNI values serving either as a binary variable (i.e., high vs. low) or a continuous parameter. The same approach was used to assess the association between PNI and disease severity, where appropriate. Because the present meta-analysis was based on observational studies, an overall OR produced using a random-effects model served as the main summary measure of effect size. We evaluated statistical heterogeneity of effect size with I^2^ statistics and defined the substantial heterogeneity as an I^2^ over 50% [25]. Sensitivity analysis through omitting one study in turn was conducted to examine the reliability and robustness of the available evidence. Potential publication bias was detected through the inspection of a funnel plot and examination with Egger’s tests regarding a particular outcome reported in 10 or more studies. The statistical analyses were conducted with the comprehensive Meta-Analysis (CMA) V3 software (Biostat, Englewood, NJ, USA).

To evaluate the accuracy of PNI for predicting mortality or disease severity, we calculated the pooled estimates of sensitivity and specificity based on the bivariate model [26]. After generation of a hierarchical summary receiver operating characteristic (hsROC) curve, the area under the curve (AUC) was used to determine the overall accuracy according to the summary receiver operating characteristic (sROC) curve. Forest plots of pooled sensitivity and specificity, sROC curve, and Deeks’ funnel plot for assessing the publication bias were generated using the MIDAS command in Stata 15 (StataCorp LLC., College Station, TX, USA). A *p* value of <0.05 was considered statistically significant in the current meta-analysis.

## 3. Results

### 3.1. Study Selection

**Figure 1:**
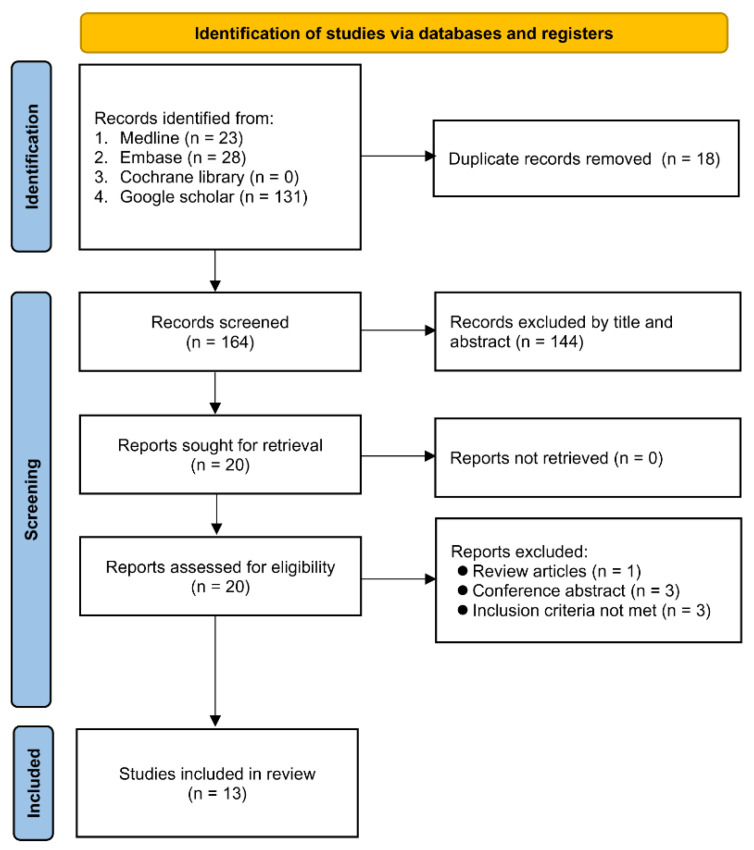
Flow chart for inclusion and exclusion.

The flow diagram depicting the process of the study selection is shown in Figure 1. Of a total of 182 records retrieved on the initial database search, 164 were preserved after excluding 18 duplicates. Following the further exclusion of 144 records during screening of their titles and abstracts, six more articles were excluded after full-text assessment because of being a review article (*n* = 1), abstract (*n* = 3), lack of available outcomes (*n* = 2), or focusing on disease progression instead of severity or mortality (*n* = 1) [27]. Finally, 13 studies involving 4204 patients published between 2020–2021 were eligible for quantitative syntheses [11,19,20,21,28,29,30,31,32,33,34,35,36].

### 3.2. Study Characteristics and Risk of Bias

The characteristics of the studies are shown in Table 1. Twelve studies reported the period for patient inclusion (i.e., all was conducted in 2020) [11,19,20,21,28,29,30,31,32,33,35,36], while one study did not provide this information [34]. The age of participants ranged from 44 to 74 years with the proportion of males between 42.3% and 61.5%. Twelve studies recruited hospitalized patients [11,19,20,21,28,29,30,31,33,34,35,36], while one study focused on patients admitted to the intensive care unit (ICU) [32]. The number of patients in all studies was over 100 with a range between 111 and 748. The included studies were conducted in three countries, including Turkey (six studies) [19,20,28,29,32,33], China (six studies) [11,21,30,31,34,35], and Iran (one study) [36]. PNI was calculated using the following equation: [(10 × serum albumin (g/dL)) + (0.005 × total lymphocyte count)] in all studies, as previously reported [37].

**Figure 2:**
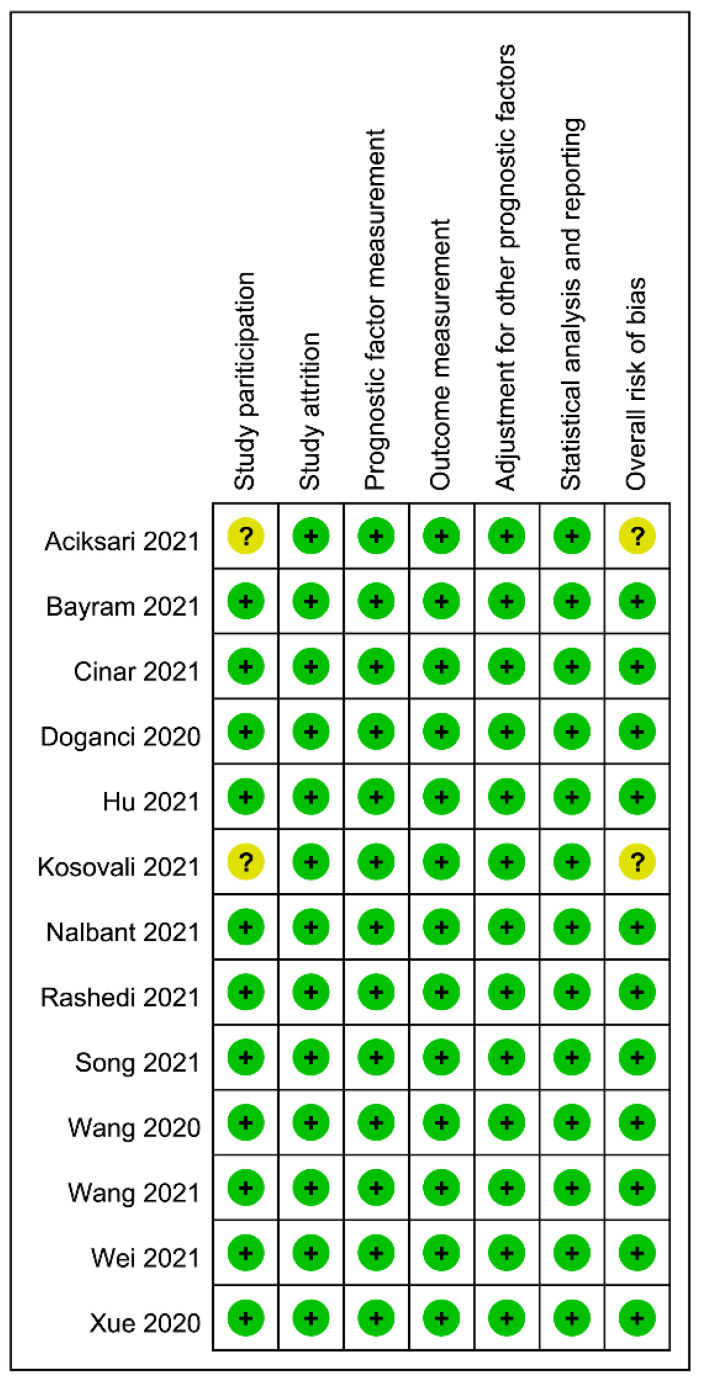
Risks of bias assessed according to the Quality in Prognostic Studies (QUIPS) tool [11,19,20,21,28,29,30,31,32,33,34,35,36].

The risks of the bias evaluated by the QUIPS tool are summarized in Figure 2. The risk of the bias of study participation in two studies was considered unclear because of the inclusion of patients with severe COVID-19 [28] or those admitted to ICU [32]. The other studies demonstrated a low risk of bias in all domains [11,19,20,21,29,30,31,33,34,35,36]. The overall risk of bias was considered to be low in the majority (84.6%) of studies.

### 3.3. Data Analysis

#### 3.3.1. Primary Outcome—Association of PNI with Mortality

The association between the risk of mortality and PNI was investigated in three different approaches; while seven studies compared the PNI between the mortality and non-mortality groups, the correlation between PNI and the risk of mortality was assessed with the former serving either as a continuous parameter or as a binary variable (i.e., high vs. low) in seven and six studies, respectively.

**Figure 3:**
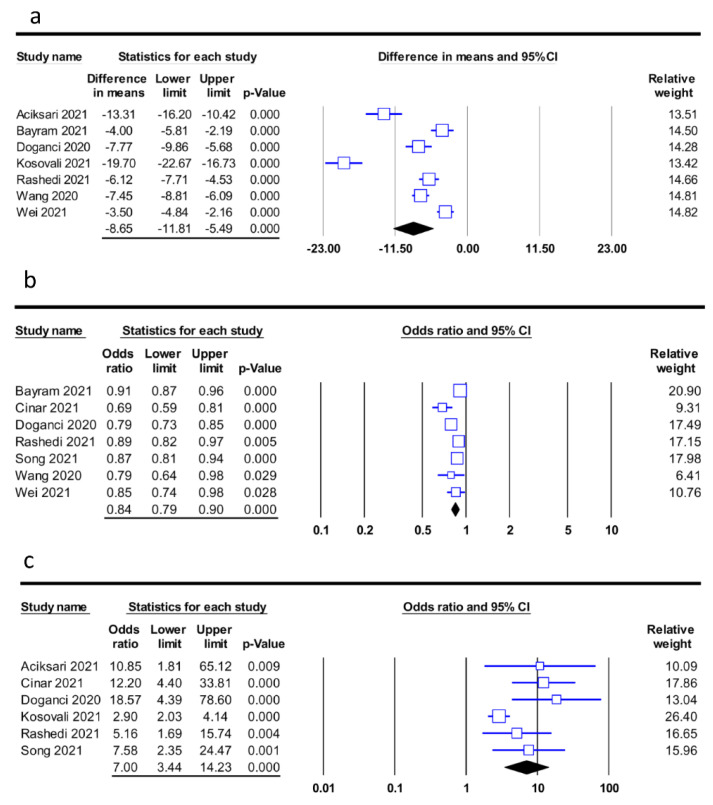
(**a**) Forest plot comparing the prognostic nutritional index (PNI) between mortality and non-mortality groups, showing a lower mean PNI in the mortality group compared to the non-mortality group (MD: −8.65, 95% CI: −11.81 to −5.49, *p* < 0.001, I^2^ = 95.3%) [19,21,28,29,32,34,36], (**b**) Forest plot demonstrating a negative correlation between risk of mortality and PNI as a continuous parameter (odds ratio: 0.84, 95% CI: 0.79 to 0.9, *p* < 0.001, I^2^ = 67.3%) [11,19,20,21,29,34,36], and (**c**) Forest plot showing a negative association between risk of mortality and PNI as a binary parameter (odds ratio: 7, 95% CI: 3.44 to 14.23, *p* < 0.001, I^2^ = 65.7%) [11,20,28,29,32,36]. CI, confidence interval.

First, based on the PNI in the mortality and non-mortality groups in seven studies [19,21,28,29,32,34,36], the merged results revealed a lower mean PNI among patients in the mortality group (MD: −8.65, 95% CI: −11.81 to −5.49, *p* < 0.001, I^2^ = 95.3%) compared to that in the non-mortality group (Figure 3a). Second, when PNI was used as a continuous measure to predict the risk of mortality in seven studies [11,19,20,21,29,34,36], the pooled results demonstrated that a higher PNI was related to a lower risk of mortality (OR: 0.84, 95% CI: 0.79 to 0.9, *p* < 0.001, I^2^ = 67.3%) (Figure 3b) with the demonstration of a correlation between an additional increase in one unit in PNI and an 16% decrease in the odds of mortality. Third, an analysis of the link between PNI as a binary variable (i.e., high vs. low) and the risk of mortality in six studies [11,20,28,29,32,36] also revealed an association of a lower PNI with a higher mortality risk (OR: 7, 95% CI: 3.44 to 14.23, *p* < 0.001, I^2^ = 65.7%) (Figure 3c). A sensitivity analysis supported the consistency of the results from the three approaches.

#### 3.3.2. Secondary Outcome—Association of PNI with Disease Severity

**Figure 4:**
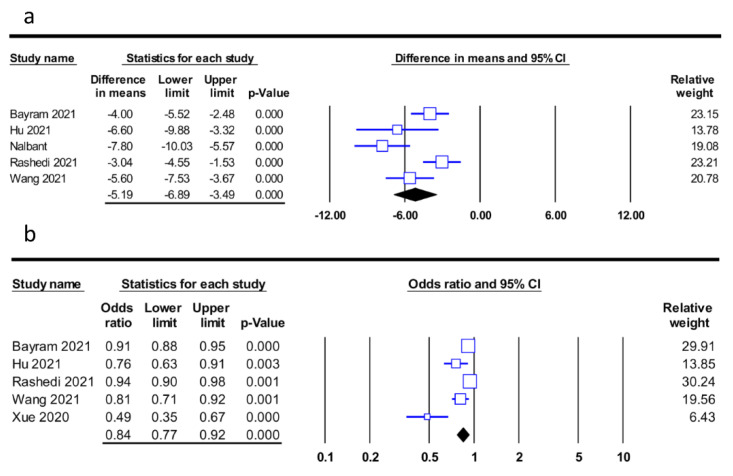
(**a**) Forest plot comparing the prognostic nutritional index (PNI) between severity and non-severity groups, showing a lower PNI in the severity group compared to the non-severity group (MD:−5.19, 95% CI: −6.89 to −3.49, *p* < 0.001, I^2^ = 73.4%) [19,21,33,35,36], and (**b**) Forest plot demonstrating a negative association between disease severity and PNI as a continuous variable (odds ratio: 0.84, 95% CI: 0.77 to 0.92, *p* < 0.001, I^2^ = 83%) [19,30,31,35,36]. CI, confidence interval.

The merged results from five studies with information on PNI in the severity and non-severity groups [19,21,33,35,36] demonstrated a lower mean PNI among patients in the severity group (MD: −5.19, 95% CI: −6.89 to −3.49, *p* < 0.001, I^2^ = 73.4%) compared to that in the non-severity group (Figure 4a). Using another approach, in which PNI served as a continuous measure to predict disease severity, the pooled results from five studies [19,30,31,35,36] also demonstrated a negative correlation between PNI and disease severity (OR: 0.84, 95% CI: 0.77 to 0.92, *p* < 0.001, I^2^ = 83%) (Figure 4b). The sensitivity analysis demonstrated the robustness of the results from the two approaches.

#### 3.3.3. The Use of PNI for Predicting Mortality and Disease Severity: Pooled Estimates of Sensitivity/Specificity and sROC

**Figure 5:**
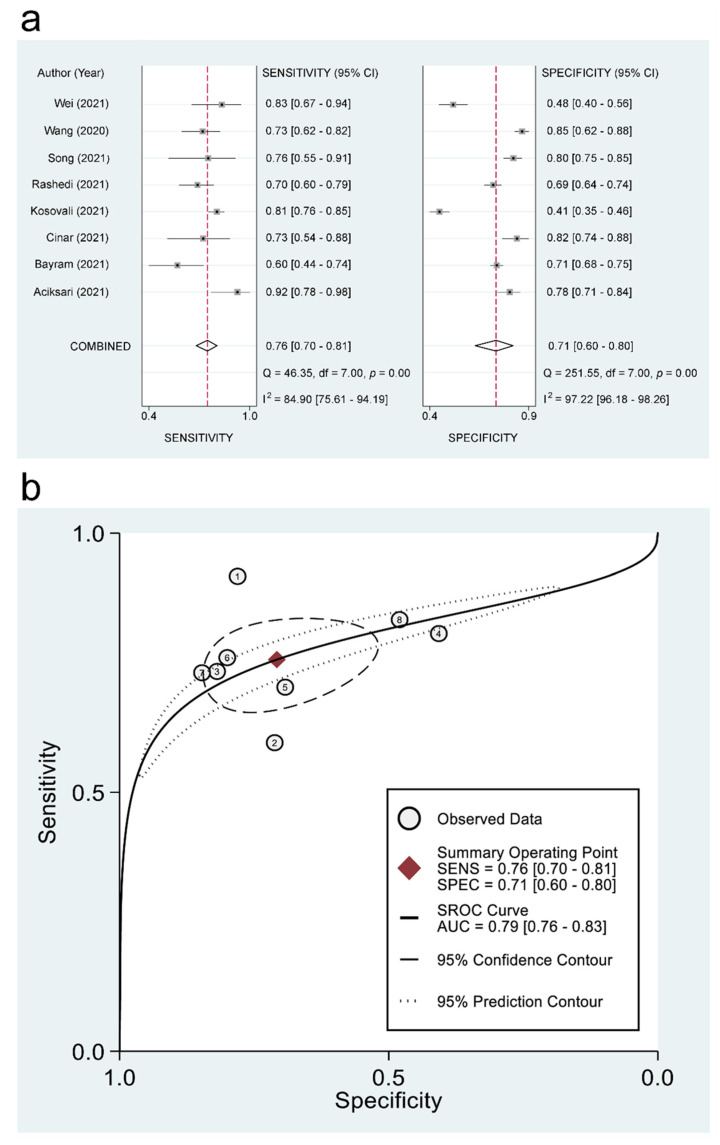
(**a**) Forest plots comparing the sensitivity and specificity of using prognostic nutritional index (PNI) for predicting in-hospital mortality in patients with COVID-19 among the included studies [11,19,20,21,28,32,34,36], and (**b**) hierarchical summary receiver operating characteristic (hsROC) curves of using PNI for the prediction of in-hospital mortality in patients with COVID-19. SENS: Sensitivity, SPEC: Specificity, SROC: Summary receiver operating characteristic, and AUC: Area under the curve.

**Figure 6:**
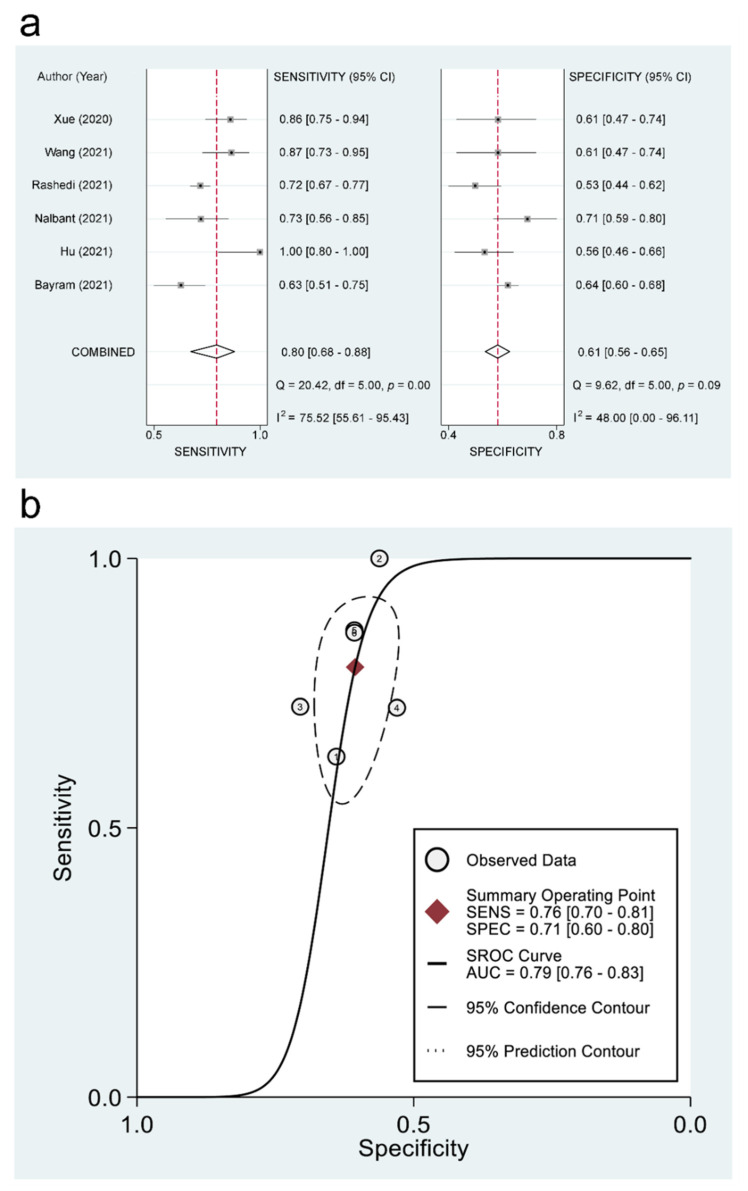
(**a**) Forest plots comparing the sensitivity and specificity of applying prognostic nutritional index (PNI) to the prediction of disease severity in patients with COVID-19 across the included studies [19,30,31,33,35,36], and (**b**) hierarchical summary receiver operating characteristic (hsROC) curves of using PNI for predicting disease severity in patients with COVID-19. SENS: Sensitivity, SPEC: Specificity, SROC: Summary receiver operating characteristic, and AUC: Area under the curve.

The pooled sensitivity and specificity of using PNI for the prediction of mortality were 0.76 (95% CI = 0.7–0.81; I^2^ = 84.9%) and 0.71 (95% CI = 0.6–0.8; I^2^ = 97.22%), respectively (Figure 5a). The linear regression for sROC generated after mathematical manipulation of true and false positivity (1-specificity) of each study revealed an AUC of 0.79 (95% CI = 0.76–0.83) (Figure 5b). Deeks’ Funnel Plot Asymmetry test showed no significant publication bias (*p* = 0.07). Regarding the use of PNI for the prediction of disease severity, pooled sensitivity and specificity were 0.8 (95% CI = 0.68–0.88; I^2^ = 75.52%) and 0.61 (95% CI = 0.56–0.65; I^2^ = 48%), respectively (Figure 6a). The linear regression for sROC demonstrated an AUC of 0.65 (95% CI = 0.61–0.69) (Figure 6b). Deeks’ Funnel Plot Asymmetry test indicated significant publication bias (*p* = 0.01).

## 4. Discussion

With the staggering increase in the number of patients diagnosed with COVID-19, the minimization of disease mortality and severity as well as a rational allocation of medical resources have become priorities in medical systems worldwide. Identification of patients at high risks of mortality and complications can guide medical decisions to facilitate timely implementation of individualized therapeutic strategies [38]. Our results not only supported the use of PNI as a predictor of mortality with a pooled sensitivity of 0.76 and specificity of 0.71 (AUC of sROC: 0.79) but also demonstrated a negative association between PNI and disease severity in patients with COVID-19. Taking into consideration the simplicity of calculation, our findings suggested that PNI may be a cost-effective indicator for medical resource allocation during the pandemic.

Regarding the factors associated with COVID-19 disease severity and mortality, a previous meta-analysis has underscored an increased susceptibility to severe diseases in those with cardiovascular diseases as well as those with chronic respiratory and renal illnesses [39,40]. Other reported indicators of disease progression and mortality also included serological biomarkers such as C-reactive protein (CRP), neutrophil–lymphocyte ratio (NLR), ferritin, troponin, and lymphocyte count [41,42]. On the other hand, although several studies demonstrated an association between a poor nutritional status and an elevated risk of in-hospital death in patients infected with COVID-19 [11,12], the pooled evidence between the PNI and prognosis of COVID-19 was rarely investigated. Our finding demonstrated that a low PNI correlated with a seven-fold increased risk of mortality in hospitalized patients with COVID-19.

There were several potential confounding factors for the current investigation. Because an alleviation of disease severity and mortality through an intensive COVID-19 vaccination program may be a confounder of our outcomes, the exclusive inclusion of studies conducted in 2020 when the vaccination rates in the studied countries were still low may help in minimizing the impact of this factor in the current meta-analysis. Besides vaccination, the overall mortality of COVID-19 has also been demonstrated to be different between men and women, with the former being 2.3 times higher than the latter. Moreover, ethnically, the mortality and disease severity rates have been reported to be lower in Asians, as compared with other populations [43,44]. Therefore, in addition to the known influences of gender and geographical location on immune responses (e.g., cytokine expressions) [6], the inclusion of studies from a limited number of countries (e.g., China and Turkey) with a comparable proportion of males and females in most studies (no relevant information in one study) may offer a high homogeneity to minimize the related biases in the current meta-analysis.

In an attempt to identify prognostic markers for COVID-19 severity and mortality, a previous study suggested a better predictive outcome when combining indicators of inflammation with those of nutritional status than that from inflammation biomarkers alone [31]. The association of malnutrition with a poor prognosis in patients with COVID-19 has been reflected in the finding of a previous study, in which patients with COVID-19 who were admitted to ICU were found to be at risk for moderate (69.9%) to severe (12.3%) malnutrition [12]. Several factors have been reported to contribute to malnutrition in patients infected with COVID-19, including elevated catabolism from fever and exertion of respiratory muscles as well as endocrinological perturbations that lead to accelerated gluconeogenesis, protein breakdown, and lipid oxidation [11]. PNI, which is readily obtainable through routine peripheral blood examinations, comprises both nutritional and inflammatory components to serve this purpose. Interestingly, although most studies in the current meta-analysis enrolled non-critical hospitalized patients, we still found a significantly negative association between PNI and prognosis that may suggest its usefulness as an early marker of disease progression before an overt deterioration of nutritional status.

The finding of a significant negative correlation between PNI and COVID-19 severity despite the adoption of different definitions for disease severity in our included studies highlighted a potential role of PNI in COVID-19 severity prediction. With an explosive increase in the number of patients with a confirmed COVID-19 diagnosis, patient triage is of the utmost importance. As the symptoms of COVID-19 vary widely, ranging from no symptoms to critical illness requiring urgent medical attention [45,46], an accurate prediction of disease progression is essential to guiding medical resource allocation to reduce mortality [47]. For instance, assigning patients with a relatively minor condition (e.g., mild pneumonia) to ambulatory care and those with a severe disease to the intensive care unit (ICU) [48]. In countries where medical resources are limited, a simple feasible indicator of disease severity may be critical for the effective allocation of medical supplies and manpower.

Notwithstanding the known adverse impact of malnutrition on disease progression and mortality of COVID-19, age may be a significant confounder. A large-scale prospective cohort study on 20,133 inpatients contracting COVID-19 identified advanced age and obesity as independent risk factors of COVID-19-related mortality [49]. Besides, the susceptibility to malnutrition in older people has been demonstrated in previous studies that exhibited a prevalence of malnutrition as high as 35–65% in aged hospitalized patients and 25–60% in older institutionalized adults [50,51]. These findings, together with the elevated mortality rate in the aged population infected with COVID-19, underscored the importance of age as a confounder factor in the present study. Nevertheless, a previous study has still demonstrated PNI as an independent predictor of COVID-19 severity after adjustment for gender, body mass index, and age [35], highlighting the applicability of this indicator in different clinical scenarios.

Although our results demonstrated robust evidence on the negative association of PNI with mortality and disease severity, several issues remain to be clarified. First, as a variety of vaccines are currently available, the correlation between PNI and prognosis in patients receiving vaccine prophylaxis should be re-evaluated. Second, as malnutrition may be a modifiable risk factor, the impacts of nutrition supplementation on mortality and disease severity in patients with COVID-19 need to be assessed. Third, although PNI has previously been used to predict the prognostic outcomes in patients with various cancers [52,53], our included study did not specifically focus on patients with cancer. Therefore, further studies are required to investigate the predictive value of PNI regarding mortality and disease severity in this subgroup of patients after being infected with COVID-19.

There are several limitations in the current study. First, the major shortcoming of our meta-analysis was the retrospective nature of data collection that rendered our results susceptible to various confounding factors. For instance, no information about viral load and treatment options, which are known determinants of the duration of hospital stay and disease severity, was available for analysis [54]. Second, although previous studies reported a superior accuracy of mortality prediction in patients with COVID pneumonia by using a combination of different prediction indices compared to the use of a single indicator [31,55], our meta-analysis could not address this issue. In addition, we also did not compare the efficacy of PNI in mortality prediction with that of other predictive tools. Third, considering the significant differences in mortality among countries and phases of the pandemic [4], our inclusion of studies mainly from China and Turkey may limit the extrapolation of our findings to other ethnic groups and geographical locations. Fourth, because previous pooled evidence has already suggested associations of other confounding factors (e.g., age and comorbidities) with mortality and disease severity in patients contracting COVID-19, the predictive values of such confounders were not evaluated in the present study. In addition, although some other health-related indicators such as history of alcohol consumption, smoking, physical activities, sleeping, working environment and social routine among hospitalized patients could also have influenced our study outcomes, there were only three studies that included information about smoking [11,20,35], in which only two mentioned alcohol consumption [11,20]. Moreover, the lack of data about quantity and duration in those studies precluded the conduction of a subgroup analysis to elucidate the significance of their effects on our results. Finally, our results may be biased by variations in the definitions of disease severity and cut-off values of PNI for mortality prediction among our included studies.

## 5. Conclusions

In conclusion, this meta-analysis of 13 cohort retrospective studies on hospitalized patients with COVID-19 supported the use of a prognostic nutritional index as a promising index in predicting mortality and disease severity. Further large-scale studies are warranted to investigate the potential benefits of incorporating this index into clinical practice to improve prognostic outcomes in patients diagnosed with COVID-19.

## Figures and Tables

**Table 1:** Characteristics of studies (*n* = 13).

Studies	Patient Enrollment Period (2020)	Age (Years)	Male (%)	Patient Number (*n* = 4204)	Definition of Severity	Outcomes	Country
Aciksari 2021	March–August ^†^	60	53	223	a	Mortality/severity	Turkey
Bayram 2021	September–December	74 vs. 61	54.8	748	ICU admission	Mortality/severity	Turkey
Cinar 2021	March–August	62 vs. 50	59.2	196	NA	Mortality	Turkey
Doganci 2020	March–May	57	50	397	NA	Mortality	Turkey
Hu 2021	January–February	44	55.7	122	a	Severity	China
Kosovali 2021	March–July ^¶^	69	54.9	690	NA	Mortality	Turkey
Nalbant 2021	January–April	58 vs. 70	50.8	118	ICU admission	Severity	Turkey
Rashedi 2021	February–November	61	61.5	504	b	Mortality/severity	Iran
Song 2021	January–May ^§^	58	52.5	295	a	Mortality/severity	China
Wang 2020	January–February	58	45.8	450	NA	Mortality	China
Wang 2021	January–March	65 vs. 49	42.3	111	c	Severity	China
Wei 2021	NA ^§^	74 vs. 55	49.2	236	d	Mortality/severity	China
Xue 2020	February–March	62	56.1	114	a	Severity	China

^†^ severe COVID patients; ^¶^ patients admitted to intensive care units; ^§^ multicenter studies; PNI: prognostic nutritional index; AUC: area under curve; a Guidance for Corona Virus Disease 2019 (7th edition) by the National Health Commission of China; b Severe disease was ascertained as patients with one of the following criteria: dyspnea, septic shock, respiratory failure, oxygen saturation ≤ 93% or >50% lung involvement on imaging, or multiple organ dysfunction/failure; c National Health Commission Guideline on the Management Of Novel Coronavirus Pneumonia; d American Thoracic Society guidelines for community-acquired pneumonia; ICU: intensive care unit; NA: not available.

## Data Availability

Not applicable.

## Supplementary Materials

The following supporting information can be downloaded at: https://www.mdpi.com/article/10.3390/diagnostics12071515/s1, Table S1: Search strategies for medline.

## References

[1] Chang D., Chang X., He Y., Tan K.J.K. (2022). The determinants of COVID-19 morbidity and mortality across countries. Sci. Rep..

[2] Guan W.J., Ni Z.Y., Hu Y., Liang W.H., Ou C.Q., He J.X., Liu L., Shan H., Lei C.L., Hui D.S.C. (2020). Clinical Characteristics of Coronavirus Disease 2019 in China. N. Engl. J. Med..

[3] Huang C., Wang Y., Li X., Ren L., Zhao J., Hu Y., Zhang L., Fan G., Xu J., Gu X. (2020). Clinical features of patients infected with 2019 novel coronavirus in Wuhan, China. Lancet.

[4] Reddy S.G.K., Mantena M., Garlapati S.K.P., Manohar B.P., Singh H., Bajwa K.S., Tiwari H. (2021). COVID-2019-2020-2021: Systematic Review and Meta-Analysis. J. Pharm. Bioallied Sci..

[5] Li L.Q., Huang T., Wang Y.Q., Wang Z.P., Liang Y., Huang T.B., Zhang H.Y., Sun W., Wang Y. (2020). COVID-19 patients’ clinical characteristics, discharge rate, and fatality rate of meta-analysis. J. Med. Virol..

[6] Hu H., Pan H., Li R., He K., Zhang H., Liu L. (2022). Increased Circulating Cytokines Have a Role in COVID-19 Severity and Death With a More Pronounced Effect in Males: A Systematic Review and Meta-Analysis. Front. Pharmacol..

[7] Zhu N., Zhang D., Wang W., Li X., Yang B., Song J., Zhao X., Huang B., Shi W., Lu R. (2020). A Novel Coronavirus from Patients with Pneumonia in China, 2019. N. Engl. J. Med..

[8] Hajiasgharzadeh K., Jafarlou M., Mansoori B., Dastmalchi N., Baradaran B., Khabbazi A. (2022). Inflammatory reflex disruption in COVID-19. Clin. Exp. Neuroimmunol..

[9] Lai C.C., Liu Y.H., Wang C.Y., Wang Y.H., Hsueh S.C., Yen M.Y., Ko W.C., Hsueh P.R. (2020). Asymptomatic carrier state, acute respiratory disease, and pneumonia due to severe acute respiratory syndrome coronavirus 2 (SARS-CoV-2): Facts and myths. J. Microbiol. Immunol. Infect..

[10] Laino M.E., Ammirabile A., Lofino L., Lundon D.J., Chiti A., Francone M., Savevski V. (2022). Prognostic findings for ICU admission in patients with COVID-19 pneumonia: Baseline and follow-up chest CT and the added value of artificial intelligence. Emerg. Radiol..

[11] Song F., Ma H., Wang S., Qin T., Xu Q., Yuan H., Li F., Wang Z., Liao Y., Tan X. (2021). Nutritional screening based on objective indices at admission predicts in-hospital mortality in patients with COVID-19. Nutr. J..

[12] Alikiaii B., Heidari Z., Fazeli A., Rahimi Varposhti M., Moradi Farsani D., Fattahpour S., Rafiee S., Bagherniya M. (2021). Evaluation of the effectiveness of the Nutritional Risk Screening System 2002 (NRS-2002) in COVID-19 patients admitted to the intensive care unit. Int. J. Clin. Pract..

[13] Pinato D.J., North B.V., Sharma R. (2012). A novel, externally validated inflammation-based prognostic algorithm in hepatocellular carcinoma: The prognostic nutritional index (PNI). Br. J. Cancer.

[14] Wang C., He W., Yuan Y., Zhang Y., Li K., Zou R., Liao Y., Liu W., Yang Z., Zuo D. (2020). Comparison of the prognostic value of inflammation-based scores in early recurrent hepatocellular carcinoma after hepatectomy. Liver Int..

[15] Peng J.C., Nie F., Li Y.J., Xu Q.Y., Xing S.P., Gao Y. (2022). Prognostic Nutritional Index as a Predictor of 30-Day Mortality Among Patients Admitted to Intensive Care Unit with Acute Exacerbation of Chronic Obstructive Pulmonary Disease: A Single-Center Retrospective Cohort Study. Med. Sci. Monit..

[16] Yao C., Liu X., Tang Z. (2017). Prognostic role of neutrophil-lymphocyte ratio and platelet-lymphocyte ratio for hospital mortality in patients with AECOPD. Int. J. Chron. Obstruct. Pulmon. Dis..

[17] Mehta P., McAuley D.F., Brown M., Sanchez E., Tattersall R.S., Manson J.J. (2020). COVID-19: Consider cytokine storm syndromes and immunosuppression. Lancet.

[18] Vong T., Yanek L.R., Wang L., Yu H., Fan C., Zhou E., Oh S.J., Szvarca D., Kim A., Potter J.J. (2022). Malnutrition Increases Hospital Length of Stay and Mortality among Adult Inpatients with COVID-19. Nutrients.

[19] Bayram M., Yildirim O., Ozmen R.S., Soylu B., Dundar A.S., Koksal A.R., Ekinci I., Akarsu M., Tabak O. (2021). Prognostic Nutritional Index and CRP, age, platelet count, albumin level score in predicting mortality and intensive care unit admission for COVID-19. Biomark. Med..

[20] Çınar T., Hayıroğlu M.İ., Çiçek V., Kılıç Ş., Asal S., Yavuz S., Selçuk M., Yalçınkaya E., Keser N., Orhan A.L. (2021). Is prognostic nutritional index a predictive marker for estimating all-cause in-hospital mortality in COVID-19 patients with cardiovascular risk factors?. Heart Lung.

[21] Wang R., He M., Yin W., Liao X., Wang B., Jin X., Ma Y., Yue J., Bai L., Liu D. (2020). The Prognostic Nutritional Index is associated with mortality of COVID-19 patients in Wuhan, China. J. Clin. Lab. Anal..

[22] Gómez C.A., Sun C.K., Tsai I.T., Chang Y.P., Lin M.C., Hung I.Y., Chang Y.J., Wang L.K., Lin Y.T., Hung K.C. (2021). Mortality and risk factors associated with pulmonary embolism in coronavirus disease 2019 patients: A systematic review and meta-analysis. Sci. Rep..

[23] Hayden J.A., van der Windt D.A., Cartwright J.L., Côté P., Bombardier C. (2013). Assessing bias in studies of prognostic factors. Ann. Intern. Med..

[24] Hung K.C., Wang L.K., Lin Y.T., Yu C.H., Chang C.Y., Sun C.K., Chen J.Y. (2022). Association of preoperative vitamin D deficiency with the risk of postoperative delirium and cognitive dysfunction: A meta-analysis. J. Clin. Anesth..

[25] Higgins J.P., Thompson S.G., Deeks J.J., Altman D.G. (2003). Measuring inconsistency in meta-analyses. BMJ.

[26] Takwoingi Y., Riley R.D., Deeks J.J. (2015). Meta-analysis of diagnostic accuracy studies in mental health. Evid. Based Ment. Health.

[27] Li Y., Li H., Song C., Lu R., Zhao Y., Lin F., Han D., Chen L., Pan P., Dai M. (2021). Early Prediction of Disease Progression in Patients with Severe COVID-19 Using C-Reactive Protein to Albumin Ratio. Dis. Markers.

[28] Açıksarı G., Koçak M., Çağ Y., Altunal L.N., Atıcı A., Çelik F.B., Bölen F., Açıksarı K., Çalışkan M. (2021). Prognostic value of inflammatory biomarkers in patients with severe COVID-19: A single-center retrospective study. Biomark. Insights.

[29] Doganci S., Ince M., Ors N., Yildirim A., Sir E., Karabacak K., Eksert S., Ozgurtas T., Tasci C., Dogan D. (2020). A new COVID-19 prediction scoring model for in-hospital mortality: Experiences from Turkey, single center retrospective cohort analysis. Eur. Rev. Med. Pharmacol. Sci..

[30] Wang Z.-H., Lin Y.-W., Wei X.-B., Li F., Liao X.-L., Yuan H.-Q., Huang D.-Z., Qin T.-H., Geng H., Wang S.-H. (2021). Predictive value of prognostic nutritional index on COVID-19 severity. Front. Nutr..

[31] Xue G., Gan X., Wu Z., Xie D., Xiong Y., Hua L., Zhou B., Zhou N., Xiang J., Li J. (2020). Novel serological biomarkers for inflammation in predicting disease severity in patients with COVID-19. Int. Immunopharmacol..

[32] Kosovali B.D., Kucuk B., Balkiz Soyal O., Mehmet Mutlu N. (2021). Can prognostic nutritional index predict mortality in intensive care patients with COVID-19?. Int. J. Clin. Pract..

[33] Nalbant A., Demirci T., Kaya T., Aydın A., Altındiş M., Güçlü E. (2021). Can prognostic nutritional index and systemic immune-inflammatory index predict disease severity in COVID-19?. Int. J. Clin. Pract..

[34] Wei W., Wu X., Jin C., Mu T., Gu G., Min M., Mu S., Han Y. (2021). Predictive significance of the prognostic nutritional index (PNI) in patients with severe COVID-19. J. Immunol. Res..

[35] Hu X., Deng H., Wang Y., Chen L., Gu X., Wang X. (2021). Predictive value of the prognostic nutritional index for the severity of coronavirus disease 2019. Nutrition.

[36] Rashedi S., Keykhaei M., Pazoki M., Ashraf H., Najafi A., Kafan S., Peirovi N., Najmeddin F., Jazayeri S.A., Kashani M. (2021). Clinical significance of prognostic nutrition index in hospitalized patients with COVID-19: Results from single-center experience with systematic review and meta-analysis. Nutr. Clin. Pract..

[37] Onodera T., Goseki N., Kosaki G. (1984). Prognostic nutritional index in gastrointestinal surgery of malnourished cancer patients. Nihon Geka Gakkai Zasshi.

[38] McGovern J., Al-Azzawi Y., Kemp O., Moffitt P., Richards C., Dolan R.D., Laird B.J., McMillan D.C., Maguire D. (2022). The relationship between frailty, nutritional status, co-morbidity, CT-body composition and systemic inflammation in patients with COVID-19. J. Transl. Med..

[39] Meng Y., Wang J., Wen K., Da W., Yang K., Zhou S., Tao Z., Liu H., Tao L. (2021). Clinical Features and Laboratory Examination to Identify Severe Patients with COVID-19: A Systematic Review and Meta-Analysis. Biomed. Res. Int..

[40] Cheng Y., Luo R., Wang K., Zhang M., Wang Z., Dong L., Li J., Yao Y., Ge S., Xu G. (2020). Kidney disease is associated with in-hospital death of patients with COVID-19. Kidney Int..

[41] Shi C., Wang L., Ye J., Gu Z., Wang S., Xia J., Xie Y., Li Q., Xu R., Lin N. (2021). Predictors of mortality in patients with coronavirus disease 2019: A systematic review and meta-analysis. BMC Infect. Dis..

[42] Wang Y., Zhao J., Yang L., Hu J., Yao Y. (2021). Value of the Neutrophil-Lymphocyte Ratio in Predicting COVID-19 Severity: A Meta-analysis. Dis. Markers.

[43] Tirupathi R., Muradova V., Shekhar R., Salim S.A., Al-Tawfiq J.A., Palabindala V. (2020). COVID-19 disparity among racial and ethnic minorities in the US: A cross sectional analysis. Travel. Med. Infect. Dis..

[44] Mackey K., Ayers C.K., Kondo K.K., Saha S., Advani S.M., Young S., Spencer H., Rusek M., Anderson J., Veazie S. (2021). Racial and Ethnic Disparities in COVID-19-Related Infections, Hospitalizations, and Deaths: A Systematic Review. Ann. Intern. Med..

[45] Gao Z., Xu Y., Sun C., Wang X., Guo Y., Qiu S., Ma K. (2021). A systematic review of asymptomatic infections with COVID-19. J. Microbiol. Immunol. Infect..

[46] Hu B., Guo H., Zhou P., Shi Z.L. (2021). Characteristics of SARS-CoV-2 and COVID-19. Nat. Rev. Microbiol..

[47] Attaway A.H., Scheraga R.G., Bhimraj A., Biehl M., Hatipoğlu U. (2021). Severe covid-19 pneumonia: Pathogenesis and clinical management. BMJ.

[48] Higuera-de-la-Tijera F., Servín-Caamaño A., Reyes-Herrera D., Flores-López A., Robiou-Vivero E.J.A., Martínez-Rivera F., Galindo-Hernández V., Rosales-Salyano V.H., Casillas-Suárez C., Chapa-Azuela O. (2021). The Age-AST-D Dimer (AAD) Regression Model Predicts Severe COVID-19 Disease. Dis. Markers.

[49] Docherty A.B., Harrison E.M., Green C.A., Hardwick H.E., Pius R., Norman L., Holden K.A., Read J.M., Dondelinger F., Carson G. (2020). Features of 20 133 UK patients in hospital with covid-19 using the ISARIC WHO Clinical Characterisation Protocol: Prospective observational cohort study. BMJ.

[50] Liu G., Zhang S., Mao Z., Wang W., Hu H. (2020). Clinical significance of nutritional risk screening for older adult patients with COVID-19. Eur. J. Clin. Nutr..

[51] Corish C.A., Bardon L.A. (2019). Malnutrition in older adults: Screening and determinants. Proc. Nutr. Soc..

[52] Luan C.W., Tsai Y.T., Yang H.Y., Chen K.Y., Chen P.H., Chou H.H. (2021). Pretreatment prognostic nutritional index as a prognostic marker in head and neck cancer: A systematic review and meta-analysis. Sci. Rep..

[53] Yang Y., Gao P., Song Y., Sun J., Chen X., Zhao J., Ma B., Wang Z. (2016). The prognostic nutritional index is a predictive indicator of prognosis and postoperative complications in gastric cancer: A meta-analysis. Eur. J. Surg. Oncol..

[54] Liu X., Zhou H., Zhou Y., Wu X., Zhao Y., Lu Y., Tan W., Yuan M., Ding X., Zou J. (2020). Risk factors associated with disease severity and length of hospital stay in COVID-19 patients. J. Infect..

[55] Satici M.O., Islam M.M., Satici C., Uygun C.N., Ademoglu E., Altunok İ., Aksel G., Eroglu S.E. (2022). The role of a noninvasive index ‘Spo2/Fio2’ in predicting mortality among patients with COVID-19 pneumonia. Am. J. Emerg. Med..

