# Supplementary material for: Association of Prognostic Nutritional Index with Severity and Mortality of Hospitalized Patients with COVID-19: A Systematic Review and Meta-Analysis
Source: Diagnostics (Basel). 2022 Jun 21;12(7):1515. doi: 10.3390/diagnostics12071515 (PMC9322949; doi:10.3390/diagnostics12071515)
Supplement: Supplementary file 1 [file diagnostics-12-01515-s001.zip › diagnostics-1772321-supplementary.pdf]

Association of prognostic nutritional index with severity and mortality of hospitalized patients with COVID-19: A systematic review and meta-analysis

Table S1. Search strategies for Medline.

1	("severe acute respiratory syndrome" or "coronavirus 2" or "coronavirus" or "corona virus" or "covid-19" or "nCoV" or "2019nCoV" or "Wuhan virus" or "2019-nCoV" or "SARS-CoV-2 Infection*").mp.
2	exp "COVID 19"/ or exp "SARS-CoV-2"/
3	("Prognostic nutritional index" or "PNI").mp.
4	("death" or "Mortality" or "survival" or "severity" or "prognosis" or "severity").mp.
5	exp " Mortality"/ or exp " Prognosis"/ or exp "survival"/
6	(1 or 2) and 3 and (4 or 5)
